# Conversion Surgery with HIPEC for Peritoneal Oli-gometastatic Gastric Cancer

**DOI:** 10.3390/cancers11111715

**Published:** 2019-11-02

**Authors:** Jerzy Mielko, Karol Rawicz-Pruszyński, Magdalena Skórzewska, Bogumiła Ciseł, Agnieszka Pikuła, Magdalena Kwietniewska, Katarzyna Gęca, Katarzyna Sędłak, Andrzej Kurylcio, Wojciech P. Polkowski

**Affiliations:** Department of Surgical Oncology, Medical University of Lublin, 20-080 Lublin, Poland; jerzymielko@uml.edu.pl (J.M.); magdalenaskorzewska@uml.edu.pl (M.S.); bogumilacisel@uml.edu.pl (B.C.); agnieszkapikula@uml.edu.pl (A.P.); magdalenakwietniewska@uml.edu.pl (M.K.); kasiaa.geca@gmail.com (K.G.); sedlak.katarz@gmail.com (K.S.); andrzejkurylcio@uml.edu.pl (A.K.); wojciechpolkowski@uml.edu.pl (W.P.P.)

**Keywords:** gastric cancer, peritoneal metastases, conversion therapy, CRS, HIPEC

## Abstract

Peritoneal metastases (PM) of gastric cancer (GC) are characterized by a particularly poor prognosis, with median survival time of 6 months, and virtually no 5-year survival reported. Conversion therapy for GC is defined as a surgical treatment aiming at an R0 resection after systemic chemotherapy for tumours that were originally unresectable (or marginally resectable) for technical and/or oncological reasons. The aim of the present study was to evaluate early and late outcomes in GC patients with PM who underwent the cytoreductive surgery (CRS) with hyperthermic intraperitoneal chemotherapy (HIPEC) after neoadjuvant (conversion) chemotherapy. Thirty patients with stage IV GC underwent CRS plus HIPEC. Severe grade III/IV (Clavien-Dindo classification) complications occurred in 13 (43%) patients. The Comprehensive Complication Index (CCI) ranged from 8.7 to 100 (median, 42.4). In the multivariate survival analysis, ypT2 and P3 (according to the Japanese classification of the PM severity) were favourable and adverse prognostic factors *p* = 0.031 and *o* = 0.035, respectively. Estimated 1- and 3-year survival was 73.9% and 36.6%, respectively. The median survival was 19.3 months. Conclusion: Conversion surgery, including extended gastrectomy and multi-organ resections followed by HIPEC performed after systemic chemotherapy therapy for GC with PM is justified in downstaged patients with ypT2 and limited (less than P3) PM.

## 1. Introduction

The problem of synchronous peritoneal metastases (PM) in gastric cancer (GC) affects about 5% to 30% of patients, whereas the incidence of peritoneal relapse is 46% to 54% [1,2]. Many metastasis-related factors, such as adhesion molecules, matrix proteases, and motility factors, are involved in a multistep process of the peritoneal dissemination [3]. The 5-year survival rate in the entire population of patients with GC is 15% to 20%; however, it decreases drastically with the stage of the disease. In locally advanced disease, the 5-year survival rate reaches up to 55%, but in stage IV (M1), it does not exceed 4% [4]. Peritoneal dissemination in the course of GC is characterized by a particularly poor prognosis, with median survival time of 6 months and virtually no 5-year survival reported [5]. When the presence of free cancer cells in the peritoneal fluid is found, the 5-year survival rate does not exceed 2% [6]. Risk factors for PM from primary GC include advanced tumour stage (T3/4), age < 60, female gender, poorly cohesive histological type (especially signet ring cell carcinoma), lymph node or liver metastases, angioinvasion, and the presence of malignant ascites [7].

Palliative chemotherapy only slightly improves outcome in this patient group, with a median survival of 10 months. Conversion therapy for GC is defined as a surgical treatment aiming at an R0 resection after systemic chemotherapy for tumours that were originally unresectable (or marginally resectable) for technical and/or oncological reasons [8]. The use of cytoreductive surgery (CRS) with intraperitoneal chemotherapy extends the median survival to 15 months [9]. The success of CRS is highly dependent on the extent of the resection. Only complete cytoreduction allows to achieve a median survival of 21–43 months, while non-radical resection is of questionable value, justified only in life-threating complications (bleeding, perforation). It has been proved that peritoneal cancer index (PCI) is a predictive factor for complete cytoreduction, with the best results obtained when the PCI score is ≤6 [10]. Therefore, only a selected group of GC patients with oligometastatic peritoneal disease may benefit from the use of CRS and HIPEC [11,12]. The aim of the present study was to evaluate early and late outcomes in GC patients with PM who underwent the CRS with HIPEC after neoadjuvant (conversion) chemotherapy.

## 2. Materials and Methods

### 2.1. Inclusion Criteria

From November 2010 to August 2018, out of the 300 patients with various peritoneal malignancies who were treated at the Department of Surgical Oncology of the Medical University of Lublin, 48 patients with advanced GC and PM after neoadjuvant systemic chemotherapy had been scheduled for surgery with an attempt of complete CRS with HIPEC, after signing informed consent form. Qualification for the CRS with HIPEC, conducted by the multidisciplinary team, was based on careful evaluation of the symptoms followed by physical examination of patients, endoscopic findings, imaging studies (CT scan), results of pathology subtyping, staging laparoscopy (unless earlier explorative laparotomy performed elsewhere), blood level of tumour markers, as well as protocols of previous procedures if surgical exploration was performed elsewhere. After laparotomy and PCI calculation, the CRS with HIPEC was ultimately performed in 30 patients (62.5%), whereas 18 patients (37.5%) underwent palliative surgery due to extensive peritoneal dissemination. After having institutional review board approval (Bioethical Committee of Medical University of Lublin, Ethic code: KE-0254/297/2018), data was analysed from prospectively maintained database. Overall survival (OS) was defined as the length of time from the date of CRS + HIPEC to patient’s death or last documented follow-up.

### 2.2. Evaluation of PM

For the objective evaluation of the distribution and volume of PM, a quantitative staging system of the Japanese Rules of Gastric Cancer was used [13]. In this system, macroscopic PM are classified into four categories (irrespective of peritoneal wash cytology and size of the PM): P0, P1, P2, and P3. The P0 means no macroscopic disease, P1 means PM present in the upper abdomen above the transverse colon, P2 means several countable PM in the peritoneal cavity, and P3 means numerous PM in the whole peritoneal cavity.

### 2.3. CRS

Gastrectomy with D2 or D2 plus lymphadenectomy, followed by peritonectomy were performed in all patients en block with greater and lesser omentum, spleen, and all other organs involved (tail of the pancreas, gallbladder, left liver lobe, small intestine, colon, diaphragmatic crura, ovaries in case of Krukenberg tumours). Gastrointestinal continuity after the extended gastrectomy was restored by the Roux-en-Y esophagojejunostomy, and all other anastomoses were performed between cytoreduction and HIPEC. Extensive CRS was considered when at least 3 organs were resected or at least 2 anastomoses were performed [14]. For the assessment of surgical complications, the Clavien-Dindo classification (Table 1) [15,16] and the CCI classification (Table 2) [17,18] were used. For the latter one, a literature-based cut-off point of 40 points was adopted for further statistical assessment [19].

### 2.4. HIPEC

After CRS completion, the patients underwent HIPEC. Intraperitoneal perfusions were performed using SunChip (Gamidatech^®^, Eaubonne, France). From November 2010 to December 2015, HIPEC was performed as an open procedure (Coliseum technique, Lublin, Poland) with a usage of the Münster retractor. Since December 2015, the HIPEC procedure was performed as a closed method. Two drug protocols were used, with either 30 mg Mitomycin C dissolved in 0.9% NaCl at 42 °C for 60 min, or 300 mg/m^2^ Oxaliplatin dissolved in 5% glucose at 43 °C for 30 min, with drug flow of 4–11 litres/minute. Throughout the procedure, the transoesophageal body temperature was additionally monitored. Critical hyperthermia was not reported.

### 2.5. Statistical Analysis

Statistical analysis was performed using MedCalc 19.1 (MedCalc Software, Ostend, Belgium). Descriptive statistics were reported as mean ± SD, median with minimum and maximum values or frequency with percentage. Univariate overall survival (OS) and recurrence free survival (RFS) analyses was performed with the use of the Kaplan–Meier estimation method and compared using the log-rank test, whereas Cox logistic regression model were used in multivariate OS analysis with pT and PCI as the only statistically significant variable included. Hazard ratios and the corresponding 95% confidence intervals were estimated with the use of Cox proportional-hazards models. A *p*-value < 0.05 was considered significant. Follow-up data included date of the most recent follow-up and patient’s recurrence statuses.

## 3. Results

Demographic data, clinical features, and treatment related characteristics of GC patients who underwent CRS + HIPEC are presented in Table 3. Among 30 patients included in the study, 20 were males (67%) and 10 females (33%), with median age of 55 years.

All patients underwent preoperative chemotherapy: 3 cycles of EOX regimen in 25 patients or 4 cycles of FLOT regimen in 5 patients. In 2 patients, preoperative chemotherapy was discontinued after the 1st cycle, due to recurrent upper gastrointestinal bleeding and indication for urgent surgery.

**Figure 1:**
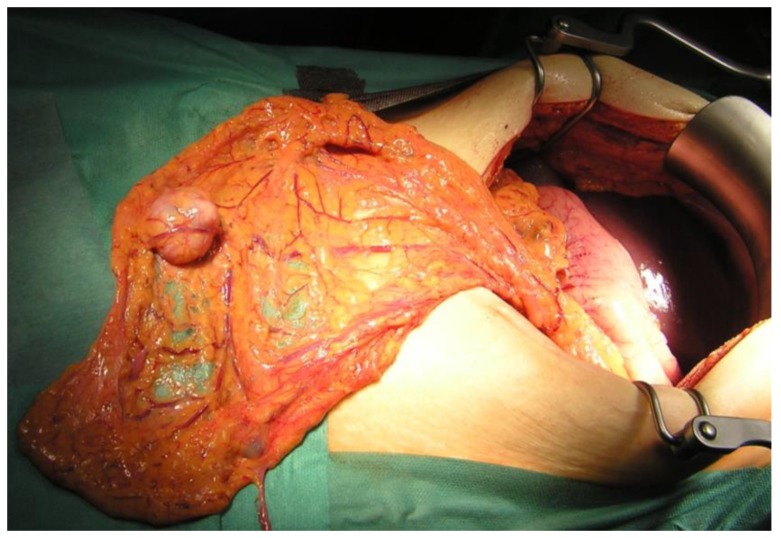
Isolated peritoneal metastasis in greater omentum (P1).

In 26 patients (87%), distant metastases were limited to the peritoneum (PM), including 6 patients (20%) with greater omentum involvement (P1), 4 patients (14%) with Krukenberg (ovarian) tumours, and 2 patients (7%) with concomitant liver metastases. In 8 patients (27%), distant (including extra-regional lymph node) metastases were found simultaneously in several locations. In 14 patients (47%), PM were directly adjacent to the serosa of the stomach, including the greater omentum (P1) (Figure 1). In 10 patients (33%), several scattered PM to the peritoneum or ovarian metastasis alone (P2), and in 6 patients (20%), numerous metastases in the distant peritoneum were found (P3). PCI ranged from 1 to 19 (median, 6; mean ± SD: 5.06 ± 4.4).

The average duration of the CRS was 221.3 min. During the HIPEC, 23 (77%) patients were perfused with the oxaliplatin, and 7 (23%) with the mitomycin C. The open HIPEC technique was performed in 22 (74%) patients whereas the closed technique in 7 (23%) patients. Extensive CRS with multiorgan resection was performed in 22 (73%) patients. Complete cytoreduction (CCR0) was achieved in 21 (70%) patients, CCR1 cytoreduction in 8 (27%) patients, and incomplete CCR2 cytoreduction in 1 (3%) patient.

Severe grade III/IV (Clavien–Dindo classification) complications occurred in 13 (43%) patients. The most common complications were wound infection (10%), postoperative gastrointestinal obstruction (10%), oedema and electrolyte disorders (6%), and postoperative bleeding (6%). Less common complications included: pancreatic fistula (3%), postoperative pancreatitis (3%), and anastamotic leakage (3%).

The CCI ranged from 8.7 to 100 (mean, 42.7; median, 42.4). The CCI above 40 was found in 12 (40%) patients. Blood transfusions were necessary in 14 (47%) patients; on average 2.1 red blood concentrate units were transfused. Seven (23%) patients required ICU stay, and the average time of hospitalization at the ICU was 3.9 days. The average time of hospitalization in the surgical ward was 15.2 days, ranging from 6 to 57 days. The 30- and 90-day postoperative death occurred in 1 (3.3%) and 2 (6.7%) patients, respectively.

In the univariate analysis of patients with GC treated with CRS plus HIPEC, ypT and PCI were significant prognostic factors (*p* = 0.03 and *p* = 0.01, respectively). Detailed results are shown in Table 4. In the multivariate analysis, ypT2 was a favourable prognostic factor, with a HR of 0.09 (CI 95%, 0.01–0.9; *p* = 0,04), whereas PCI was an adverse prognostic factor, with a HR of 1.1 (CI 95%, 1.01–1.26; *p* = 0.031)

**Figure 2:**
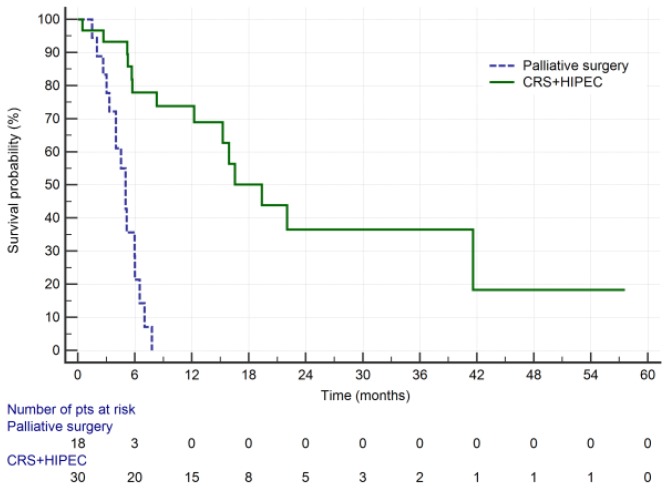
Overall survival of GC patients with PM treated with CRS + HIPEC vs. palliative surgery.

The survival curves of GC patients with PM treated with CRS + HIPEC and palliative surgery are presented in Figure 2. Median OS was significantly longer for CRS + HIPEC group compared with palliative surgery group (19.3 vs. 5 months, *p* = 0.0001, log rank test).

**Figure 3:**
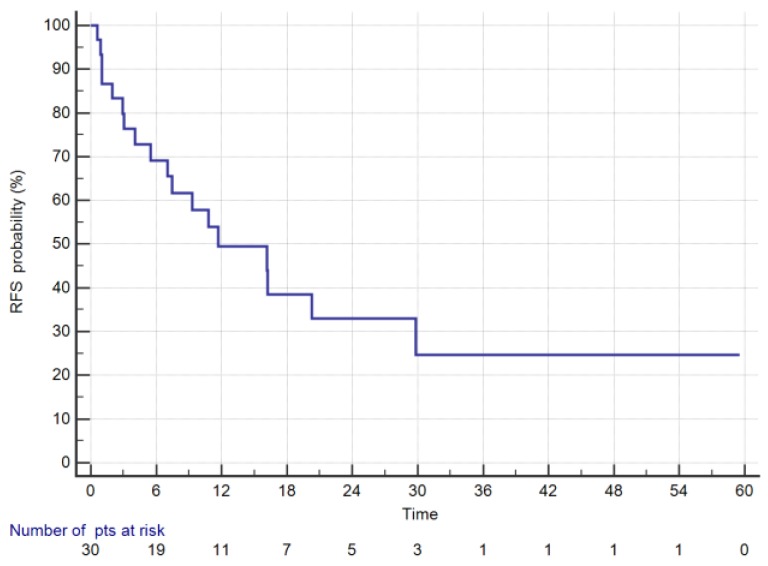
Recurrence free survival of GC patients with PM treated with CRS + HIPEC.

For CRS + HIPEC group, estimated 1- and 3-year survival was 73.9% and 36.6%, respectively. During the follow-up, 14 patients (46.7%) died, while recurrence was found in 18 patients (60%). Median follow up was 38 months (range 4–94). The date of data cut-off was 30 August 2019. Median RFS was 11,7 months (Figure 3). RFS rates at 1 and 3 years were 49.4% and 24.7%, respectively.

Patients with diffuse or mixed type GC had a median survival of 15.9 months, whereas more than half of patients with intestinal type GC remained alive under observation, and median of their survival was not reached (*p* = 0.06; log-rank test). Median survival of patients with moderately differentiated (G2) tumours was 41.5 months, while in patients with poorly differentiated (G3) tumours 12.2 months (*p* = 0.07; log-rank test).

**Figure 4:**
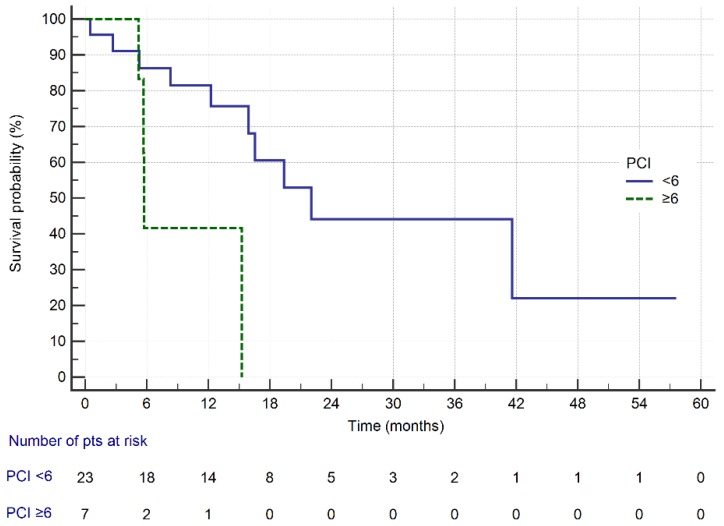
Overall survival of GC patients treated with CRS + HIPEC depending on the PCI with cut-off value of 6 (*p* = 0.01; log-rank test).

Figure 4 shows the survival curves of GC patients treated with CRS plus HIPEC depending on the median PCI score, with the cut-off value of 6. The estimated median survival in patients with PCI less than 6 was 22 months, while in patients with PCI 6 or more the median survival was 5.7 months (*p* = 0.01; log-rank test).

**Figure 5:**
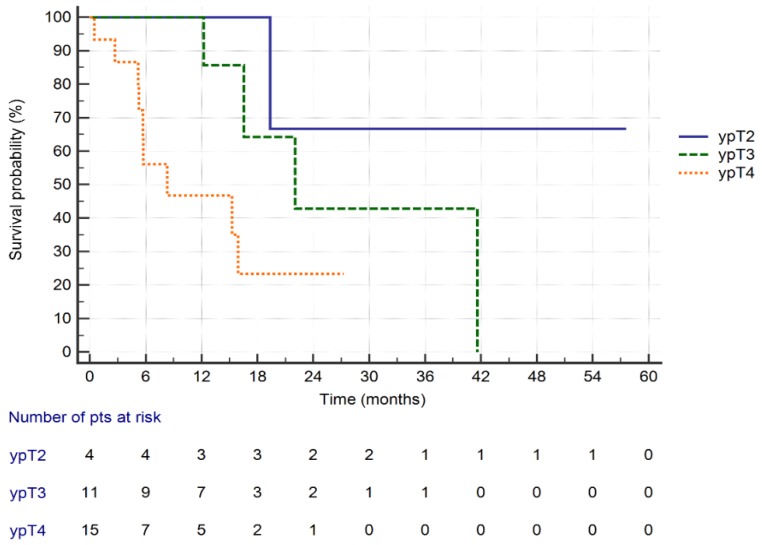
Overall survival of GC patients treated with CRS + HIPEC depending on the ypT feature (*p* = 0.03; log-rank test).

The survival curves of patients with GC treated with CRS plus HIPEC according to the ypT stage are presented in Figure 5. In patients with ypT2 tumours, median survival was not reached. In patients with the ypT3 tumours, the estimated median survival was 22 months, whereas in patients with ypT4a/b tumours, 8.2 months (*p* = 0.03; log-rank test).

In patients with different extent of PM according to the Japanese classification, the estimated median survival with P1, P2, and P3 feature was 41.5 months, 22 months, and 5.7 months, respectively (*p* = 0.07; log-rank test).

## 4. Discussion

This study evaluated the role of CRS + HIPEC by means of conversion therapy in oligometastatic GC patients. The median OS for CRS + HIPEC group was nearly 20 months compared with 5 months in palliative surgery group. PCI ≤ 6 was a significant prognostic factor, which is consistent with previously reported data [20].

REGATTA phase 3 randomized controlled trial (RCT) did not show a survival benefit in oligometastatic GC patients treated with gastrectomy plus chemotherapy versus chemotherapy alone [21]. However, the extent of lymphadenectomy in patients assigned to surgical treatment was limited to perigastric lymph nodes (D1). Neither D2 lymphadenectomy nor combined resection of adjacent organs was acceptable. Moreover, authors suggested a potential value of conversion surgery, since 5 patients initially assigned to chemotherapy alone underwent surgical treatment with curative intent due to substantial regression of the disease during chemotherapy. The AIO-FLOT3 trial was a prospective, phase 2 study that evaluated induction chemotherapy followed by surgery for GC patients with limited metastases. The median OS for patients who underwent surgical treatment was 31.3 months, compared to median OS of 15.9 months for patients who did not receive surgical intervention [22]. An ongoing prospective, randomized phase III trial (RENAISSANCE / AIO-FLOT5) addresses the potential benefits of extended surgery in GC patients with limited metastases who responded to four cycles of the FLOT [23]. As compared to our study, the RENAISSANCE (AIO-FLOT5) trial includes only localized, potentially operable peritoneal carcinomatosis stage P1 (according to classification of the Japanese Research Society for Gastric Cancer), whereas more advanced peritoneal carcinomatosis (P2/3) is an exclusion criterion.

In stage IV GC patients, surgery should only be considered as palliation of symptoms that cannot be treated endoscopically (stenting) or systematically (chemotherapy, radiotherapy), due to poor survival and high morbidity rate. On the other hand, it has been reported that CRS combined with HIPEC has the potential to control peritoneal disease in GC, with median survival of 9–12 months and 1-year survival of 36% to 48% [1,24]. Two RCTs have confirmed the clinical efficacy of CRS plus HIPEC for GC, although it was associated with high mortality and morbidity [10,25]. Nevertheless, the clinical significance of CRS plus HIPEC for GC is currently under investigation in the neoadjuvant setting, especially in the West [22]. Selected case series reporting outcome of the CRS with HIPEC with various preoperative systemic chemotherapy regimens are shown in Table 5. In this study, the estimated 1-year and 3-year survival rates were 74% and 37%, respectively. The median survival was 19 months.

A meta-analysis of 20 prospective studies involving a total of 2145 patients with advanced GC and PM showed benefit of intraperitoneal chemotherapy after R0 resection [19]. Thus, OS improves if surgery is complemented by intraperitoneal chemotherapy. However, it does not change in patients with lymph node metastases or serosal infiltration. Therefore, intraperitoneal chemotherapy is not contraindicated by lymph node involvement. Intraperitoneal chemotherapy reduces the peritoneal recurrence and occurrence of distant metastases, while increasing the postoperative complications rate. A meta-analysis of eight randomized trials which assessed the effect of HIPEC after CRS on survival, showed a significant improvement in 1-, 2- and 3-year survival rates [19]. In addition, HIPEC has a positive effect on preventing peritoneal recurrence after therapeutic resection in patients with T4 tumours. The occurrence of postoperative complications is considered an independent prognostic factor for worse survival [8]. The meta-analysis did not show a significant difference in postoperative mortality rates between HIPEC and control groups [21]. In a French, multi-institutional study, the P3, failure to perform either gastrectomy or systemic/intraperitoneal chemotherapy and age over 60 years proved to be adverse factors, resulting in poor survival [24].

In the multivariate analysis of this study, ypT and P stage (according to the Japanese classification of the PM severity) were independent prognostic factors. The ypT2 was a favourable prognostic factor, while the P3 was an unfavourable prognostic factor. The systemic treatment in combination with CRS and HIPEC showed a significant benefit in terms of survival in patients with P1 and P2 disease. Phase III studies are currently underway to determine the role of HIPEC in the surgical treatment of GC with PM [25].

This study contains certain limitations. Due to its retrospective nature, it cannot identify causation. Moreover, due to the relatively small sample size, subgroup stratification analysis might be biased.

## 5. Conclusions

Conversion surgery, including extended gastrectomy and multi-organ resections followed by HIPEC performed after systemic chemotherapy therapy for GC with PM is justified in downstaged patients with ypT2 and limited (less than P3) PM.

## Figures and Tables

**Table 1:** The Clavien-Dindo Classification.

Grade	Definition
Grade I	Any deviation from the normal postoperative course without the need for pharmacological treatment or surgical, endoscopic, and radiological interventions. Allowed therapeutic regimens are drugs as antiemetics, antipyretics, analgesics, diuretics and electrolytes and physiotherapy. This grade also includes wound infections opened at the bedside.
Grade II	Requiring pharmacological treatment with drugs other than such allowed for grade I complications. Blood transfusions and total parenteral nutrition are also included.
Grade III	Requiring surgical, endoscopic, or radiological intervention
- IIIa	Intervention not under general anaesthesia
- IIIb	Intervention under general anaesthesia
Grade IV	Life-threatening complication requiring IC/ICU-management
- IVa	single organ dysfunction
- IVb	multiorgan dysfunction
Grade V	Death of a patient

Based on: Dindo, D. et al. “Classification of surgical complications: a new proposal with evaluation in a cohort of 6336 patients and results of a survey” [16].

**Table 2:** The Comprehensive Complication Index Calculator.

Clavien–Dindo Grade *	wC **	CCI^®^ Single Value
Grade I	300	8.7
Grade II	1750	20.9
Grade IIIa	2750	26.2
Grade IIIb	4550	33.7
Grade IVa	7200	42.4
Grade IVb	8550	46.2

* Clavien–Dindo grade V always results in CCI^®^ 100. ** wC = Weight of Complication. Based on: Clavien, P.A. et al. “Comprehensive Complication Index (CCI(R)): Added Value and Clinical Perspectives 3 Years “Down the Line”” [18].

**Table 3:** Patient demographic and treatment related characteristics.

Variables		No. of Patients = 30 (%)
Age in years, mean ± SD; median, range		51 ± 11.2; 55, 28–70
Gender: male/female		20/10 (67/33 *)
Neoadjuvant chemotherapy		
	EOX	25 (83)
	FLOT	5 (17)
Pathological Assessment		
	Lauren type: intestinal/diffuse	7/23 (23/77)
	ypT2/ypT3/ypT4	4/11/15 (13/37/50)
	pN1/pN2/pN3	17/8/5 (57/27/16)
	signet-ring cells	23 (77)
	G2/G3	9/21 (30/70)
	P1/P2/P3	14/10/6 (47/33/20)
PCI, mean ± SD; median, range		5.06 ± 4.4; 6, 0–19
	<6 * PCI	23 (77)
	≥6 PCI	7 (23)
CRS time (minutes) mean ± SD; median, range		221.3 ± 58.7; 240, 90–300
Completeness Cytoreduction Score CC0/CC1, CC2		21/9 (70/30)
Extensive cytoreduction		22 (73)
Surgical Gastric procedure		
Total gastrectomy		30 (100)
Lymphadenectomy		
	D2	25 (83)
	D2+	3 (10)
	D3	2 (7)
Visceral resections		
	Cholecystecomy	7 (23)
	Appendectomy	5 (17)
	Ovariectomy	4 (13)
	Distal pancreatectomy	3 (10)
	Small bowel resection	2 (7)
	Transverse colon resection	2 (7)
	Liver metastasectomy	2 (7)
	Hysterectomy	1 (3)
Peritonectomies		
	Left diaphragm	2 (7)
	Right diaphragm	2 (7)
	Pelvic peritoneum	3 (10)
Mesenteric peritonectomy/electrovaporation		21 (70)
Intraperitoneal chemotherapy Mitomycin C/Oxaliplatin		23/7 (77/23)
HIPEC open/closed/laparoscopic		22/7/1 (74/23/3)
Postoperative complications (Clavien–Dindo Classification)		
	I	1 (3)
	II	7 (23)
	III	3 (10)
	IV	10 (33)
	V	1 (3)
Comprehensive Complication Index CCI, mean ± SD; median, range		42.7 ± 22.7; 42.4, 8.7–100
Numbers of patients requiring ICU		7 (23)
ICU stay (days) mean ± SD; median, range		3.9 ± 2.3; 4, 2–14
Hospital stay (days) mean ± SD; median, range		15.2 ± 12; 11.5, 6–57
Postoperative mortality		
	30 days	1 (3.3)
	90 days	2 (6.7)

* values in brackets are percentages.

**Table 4:** Univariate analysis of predictors for overall survival.

Variables		Overall Survival Median	*p* Value Log-Rank Test
Age (years)	<55 *	16.5	0.91
	≥55	19.3	
Gender	Male	19.3	0.7
	Female	16.5	
HIPEC protocol	MMC	8.2	0.7
	Oxaliplatin	16.5	
HIPEC technique	Closed	16.5	0.79
	Open	19.3	
CRS time (minutes)	<240 *	19.3	0.33
	≥240	16.5	
Laurén classification	Intestinal	#	0.06
	Diffuse/mixed	15.9	
Grade (histology)	G2	41.5	0.07
	G3	12.2	
ypT	pT2	#	0.03
	pT3	22.0	
	pT4a/b	8.2	
ypN	pN0	25.4	0.8
	pN+	18.1	
Peritoneal metastases classification (P)	P1	41.5	0.07
	P2	22.0	
	P3	5.7	
PCI	<6 * PCI	22.0	0.01
	≥6 PCI	5.7	
Completeness of cytoreduction score	CC0	16.5	0.1
	CC1/2	#	
Extensive cytoreduction	Normal	30.2	0.2
	Extensive	24.9	
Hospital stay (days)	<9 *	19.3	0.4
	≥9	15.9	
Postoperative complications (Clavien-Dindo Classification)	0, I, II grade	22.0	0.1
	III, IV grade	15.2	
Comprehensive Complication Index CCI	<40 *	19.3	0.28
	>40	15.2	

* median value was accepted as cut-off points. # median survival was not reached. Bold values denote statistical significance at the *p* < 0.05 level.

**Table 5:** Selected case series reporting outcome of the CRS with HIPEC with various preoperative systemic chemotherapy regimens.

Author (Year) (Ref.#)	Number of Patients	Cytostatic for HIPEC	Mortality (%)	Morbidity (%)	3-Year Survival (%)
Hall (2004) [26]	34	MMC	0	35	45
Glehen (2010) [24]	139	MMC ± Cisplatine Oxaliplatine ± Irinotecan	6.5	28	18
Yang (2011) [10]	34	MMC + Cisplatine	-	15	15
Magge (2014) [27]	23	MMC	4.3	52	18
Boerner (2016) [28]	38	Cisplatine + Doxorubicin	-	-	43
present study	30	MMC Oxaliplatine	3	43	37
